# Improved Detection of Extrapulmonary and Paucibacillary Pulmonary Tuberculosis by Xpert MTB Host Response in a Tuberculosis Low-Endemic, High-Resource Setting

**DOI:** 10.1093/infdis/jiaf110

**Published:** 2025-03-06

**Authors:** Elin Folkesson, Gabrielle Fröberg, Christopher Sundling, Thomas Schön, Erik Södersten, Judith Bruchfeld

**Affiliations:** Division of Infectious Diseases, Department of Medicine Solna, Karolinska Institutet, Stockholm, Sweden; Department of Infectious Diseases, Karolinska University Hospital, Stockholm, Sweden; Division of Infectious Diseases, Department of Medicine Solna, Karolinska Institutet, Stockholm, Sweden; Department of Clinical Microbiology, Karolinska University Hospital, Stockholm, Sweden; Division of Infectious Diseases, Department of Medicine Solna, Karolinska Institutet, Stockholm, Sweden; Department of Infectious Diseases, Karolinska University Hospital, Stockholm, Sweden; Center for Molecular Medicine, Karolinska Institutet, Stockholm, Sweden; Division of Infection and Inflammation, Department of Biomedical and Clinical Sciences, Linköping University, Linköping, Sweden; Department of Infectious Diseases, Kalmar County Hospital, Linköping University, Kalmar, Sweden; Research and Development, Cepheid AB, Solna, Sweden; Division of Infectious Diseases, Department of Medicine Solna, Karolinska Institutet, Stockholm, Sweden; Department of Infectious Diseases, Karolinska University Hospital, Stockholm, Sweden

**Keywords:** tuberculosis, extrapulmonary tuberculosis, blood based, diagnostic methods, host-related marker

## Abstract

**Background:**

The Xpert MTB Host Response (MTB-HR) assay has reached World Health Organization (WHO) test targets for pulmonary tuberculosis (PTB) with high bacillary loads. We investigated the contribution of MTB-HR as a nonsputum, near point-of-care diagnostic method in other prioritized groups, such as extrapulmonary tuberculosis (EPTB) and paucibacillary PTB.

**Methods:**

Individuals with presumed tuberculosis disease were prospectively included in Stockholm, Sweden (n = 307), and underwent MTB-HR venous and capillary testing in parallel. Clinical characterization was based on symptoms, microbiological results (microscopy, polymerase chain reaction [PCR], and culture), radiological assessment, and a panel of biochemical tests. Receiver operating characteristic analysis was performed to calculate cut-offs for maximized sensitivity and specificity, including WHO targets for screening and diagnostic tests.

**Results:**

MTB-HR performed equally well in microbiologically confirmed PTB (area under the curve [AUC], 0.84 [95% confidence interval {CI}, .78–.90]; n = 69) and EPTB (AUC, 0.82 [95% CI, .75–.90]; n = 34). Based on Youden index cut-offs, the negative predictive value (NPV) was high both in PCR-negative PTB (−1.27, NPV 94%) and in EPTB (−1.58, NPV 95%) and fulfilled the minimum target product profile sensitivity requirement for confirmed EPTB. In individuals without tuberculosis (n = 204), the majority had pulmonary infections. There was a close to perfect correlation between venous and capillary samples (*r* = 0.97, *P* < .001).

**Conclusions:**

Capillary Xpert MTB-HR improves detection of sputum PCR-negative, culture-verified PTB and is promising as a rule-out test in EPTB. MTB-HR score and bacterial burden were highly correlated. We suggest a graded MTB-HR score as more clinically relevant than a binary result.

## Teaser

In a high-resource setting, Xpert MTB-HR performs equally well in pulmonary and extrapulmonary tuberculosis (TB), matched against a complete diagnostic workup. It offers improved detection of PCR-negative pulmonary TB and disseminated TB, and is a potential rule-out test in extrapulmonary TB.

## Graphical Abstract

**Graphical Abstract:**
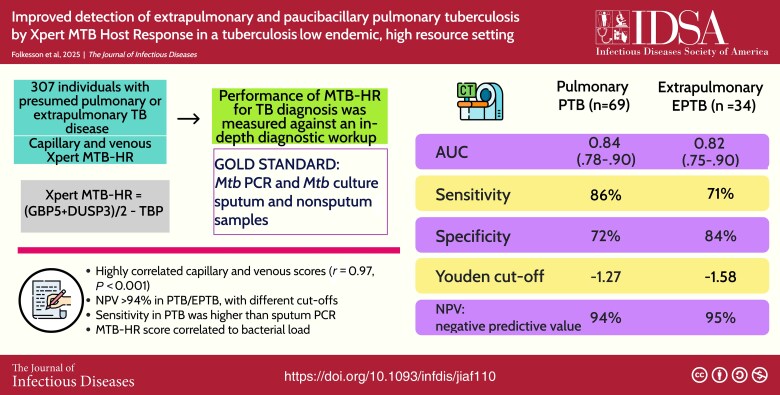
This graphical abstract is also available at Tidbit: https://tidbitapp.io/tidbits/improved-detection-of-extrapulmonary-and-paucibacillary-pulmonary-tuberculosis-by-xpert-mtb-host-response-in-a-tb-low-endemic-high-resource-setting?utm_campaign=tidbitlinkshare&utm_source=IO

Diagnostic challenges remain an obstacle toward achieving the End TB targets [1]. There is no reliable biomarker for tuberculosis (TB) disease except for microbiological detection of *Mycobacterium tuberculosis* (*Mtb*) by microscopy, polymerase chain reaction (PCR), or culture. For pulmonary TB (PTB), difficulties include obtaining sputum samples, combined with a microscopy sensitivity usually below 50% and poor access to TB healthcare and laboratory facilities [2, 3]. This contributed to an estimated diagnostic gap of 2.7 million TB cases in 2023 and insufficient microbiological confirmation (63%) among the 6.9 million reported PTB cases globally [4], with a median 2-month diagnostic delay in low- and middle-income countries [5]. Extrapulmonary TB (EPTB) diagnosis is even more challenging due to the need for invasive sample collection with a low sensitivity of current methods coupled with nonspecific clinical presentation [6–10]. Globally, EPTB constitutes 16% of reported cases with a large variation in incidence between countries, even within the European region [4, 11], likely reflecting diagnostic possibilities.

Non-sputum-based diagnostic tests, ideally for point-of-care (POC) use for rapid results, are needed to improve TB detection and reduce transmission. The World Health Organization (WHO) Target Product Profile (TPP) [12] specifies a performance minimum of a PTB screening test at 90% sensitivity and 70% specificity. For EPTB, the TPP requirements are less well-defined with a stated sensitivity of 65% for all culture-verified TB, but the importance of an improved test for EPTB is given more emphasis in the updated WHO targets for TB diagnostic tests [13]. The Cepheid Xpert MTB Host Response (MTB-HR) prototype cartridge for the GeneXpert quantitative PCR platform is the first host biomarker test for TB disease adapted for routine use to be evaluated in clinical trials. The test, which measures messenger RNA (mRNA) expression in blood, was originally based on a 3-gene signature (*GBP5*, *DUSP3*, and *KLF*) associated with PTB identified in 2016 by Sweeney and colleagues [14]. In initial assessments, the Sweeney3 signature nearly fulfilled the TPP screening test requirements for PTB [15], and in early evaluations of the MTB-HR cartridge, the area under the curve (AUC) ranged between 0.84 and 0.94 for PTB confirmed by Xpert MTB/RIF assay [16, 17].

The first multisite study in TB high-endemic countries evaluating MTB-HR reported an AUC of 0.88 for PTB set against a composite microbiological reference, whereas set against only Xpert Ultra as reference, MTB-HR fulfilled screening test requirements (AUC, 0.94; 90% sensitivity, 86% specificity) [18]. They also found that the expression of *KLF* did not separate TB disease from controls. Product development has resulted in a modified MTB-HR cartridge prototype including the gene *TBP*, intended to replace *KLF* due to a higher stability in different blood sampling methods. A recent study conducted in 6 TB-endemic countries observed an AUC of 0.89 for the modified signature (*GBP5*, *DUSP3*, *TBP*), and although the suggested cut-off value did not fulfill TPP screening targets as a rule-in test, it was concluded that MTB-HR could contribute to the diagnostic cascade due to its high negative predictive value (NPV) [19].

In this study, the aim was to assess the diagnostic performance and added value of MTB-HR in TB disease, focusing on difficult-to-diagnose paucibacillary PTB and EPTB, with an in-depth TB diagnostic workup in a high-resource, low-endemic setting used as reference.

## MATERIALS AND METHODS

### Study Population

The study was conducted at the Department of Infectious Diseases, Karolinska University Hospital, Stockholm, Sweden, a tertiary centralized referral center for investigation and treatment of TB in Stockholm County Council (population 2.4 million). The population is diverse, and most individuals with presumed and confirmed TB disease are migrants from TB-endemic countries. Patients aged ≥18 years with presumed TB disease were prospectively included between September 2019 and December 2022 during regular outpatient clinic visits and in the infectious disease wards. Patients were eligible for the study up to 72 hours from TB treatment start, based on the available data [14] on signature dynamics during treatment, combined with practical considerations to allow for patient inclusion from the inpatient wards even for weekend admissions.

### Inclusion and Exclusion Criteria

Inclusion criteria consisted of clinical suspicion of TB disease, pulmonary or extrapulmonary, with any type of patient specimen for mycobacterial analysis being prescribed; or individual newly diagnosed with TB disease initiating TB treatment. Exclusion criteria included >72 hours since initiation of treatment for TB disease; or informed consent not possible due to lack of interpreter or cognitive impairment.

### Data Collection

Demographic, structured epidemiological, and clinical information were collected in a study-specific electronic case report form (see Supplementary Material) by the attending physician. Complementary information was retrieved using the electronic chart system of Stockholm County Region public healthcare.

### Xpert MTB-HR Sampling and Analysis

Analysis was performed on-site on venous and capillary blood samples in parallel using the GeneXpert 4-module instrument. A 2 mL ethylenediaminetetraacetic acid (EDTA) tube was used for venous sampling, and 100 µL of blood was then pipetted to the test cartridge. A finger-prick was performed for the capillary sampling and blood was transferred to the test cartridge using a 100 µL minivette (Sarstedt, EDTA). The Xpert MTB-HR prototype RUO, developed and manufactured at Cepheid in Solna, Sweden, measured the individual mRNA levels of the genes *GBP5*, *DUSP3*, *TBP*, and *KLF*. The software calculated a TBP-based score (Ct^GBP5^ + Ct^DUSP3^) / 2 – Ct^TBP^, which was used throughout this study, as well as a KLF2 score (Ct^GBP5^ + Ct^DUSP3^) / 2 – Ct^KLF2^, not used in this study. In a first preliminary analysis of our data, performance of the 2 different scores was equivalent: (TBP score: AUC, 0.835 [95% confidence interval {CI}, .782–.887]; KLF2 score: AUC, 0.831 [95% CI, .779–.884]) (Supplementary Figure 1). Test data were uploaded to cloud storage and only made available after study completion.

### Mycobacterial Sampling and Diagnostic Workup

In presumed PTB, 3 sputum samples for *Mtb* analysis were collected and gastric lavage, induced sputum, and/or bronchoscopy were performed if clinically warranted. In presumed EPTB, the types of patient samples were selected according to clinical presentation (eg, aspirate, tissue biopsy, urine or other) and sputum samples taken whenever possible. Mycobacterial analysis was performed at the Karolinska University Hospital TB Laboratory by fluorescence microscopy, PCR (BD Max MDR-TB), and mycobacterial culture on liquid (BACTEC MGIT 960) and solid (Löwenstein-Jensen) medium (in-house). Positive sputum microscopy results were graded as low, medium, or high [20]. Time to culture positivity (TTP) was collected from the MGIT system. A standard biochemistry panel (erythrocyte sedimentation rate [ESR], C-reactive protein [CRP], hemoglobin, white blood cell count, neutrophil count, and albumin), QuantiFERON GOLD Plus (Qiagen), and a chest radiograph were performed. Complementary diagnostic investigations were done according to the clinician's decision.

### Follow-up

Participants were followed according to clinical routine until TB disease was confirmed or ruled out. The final diagnosis as defined in routine healthcare was verified through the electronic chart system after 6 months and at study completion. Microbiologically confirmed TB disease was defined as a positive *Mtb* PCR and/or *Mtb* culture. Clinical diagnosis of TB disease was based on a combination of symptoms, histopathological findings consistent with TB (necrotizing granuloma), radiology, and epidemiology, combined with response to TB treatment.

### Ethical Considerations

The study was granted ethical approval by the Swedish Ethical Review Board (Dnr 2019-00862) and registered with the Karolinska University Hospital clinical trial registry. Study participants received verbal and written information and provided written informed consent prior to study inclusion. Professional interpreter services were used if needed.

### Statistical Analysis

Power calculations were based on an estimated 25% TB prevalence, and an expected AUC of 0.84 for the 3-gene signature, with sensitivity 0.9 and specificity 0.47, based on original work by Sweeney et al [14]. Three hundred study participants would enable a diagnostic precision of d = 0.06 for AUC calculations. Xpert MTB-HR diagnostic performance was evaluated with receiver operating characteristic (ROC) analysis, calculating the AUC for TB disease overall, and for PTB and EPTB separately. DeLong test [21] was used to compare ROC curves.

Optimal test cut-offs were calculated using Youden index and Topleft criterion threshold [22, 23]. Sensitivity, specificity, positive predictive value, and NPV were assessed overall and in accordance with WHO TPP. Mann-Whitney and analysis of variance tests were used to compare means, and Pearson correlation was used for correlation. Statistical analyses were performed in R, SPSS (v28.0.0.0), and Jamovi (v2.4.12) software [24].

## RESULTS

### Study Population Characteristics

**Figure 1.**
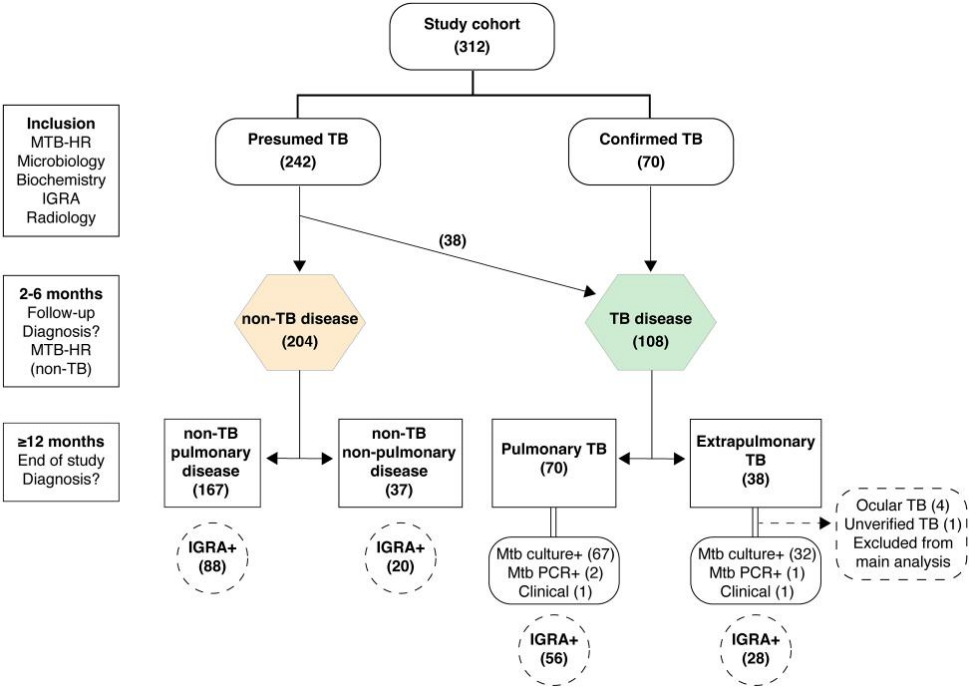
Flowchart describing patient cohort in the evaluation of Xpert MTB Host Response (MTB-HR) as a diagnostic tool for tuberculosis (TB) disease. Patients’ status at inclusion and final diagnosis: TB disease and non-TB disease including number of participants with positive interferon-gamma release assay (IGRA) status. TB and non-TB are further divided into pulmonary and extrapulmonary/nonpulmonary disease, with basis for TB diagnosis indicated. Four individuals with a clinical TB diagnosis (3 with ocular TB and 1 with presumed abdominal TB with negative mycobacterial culture and concurrent peritoneal carcinosis) were excluded from the main analysis.

In total, 312 (48% female) individuals with presumed TB were included where TB disease was diagnosed in 108 (42% female) study participants (PTB, n = 70; EPTB, n = 38; Figure 1). Most participants were immigrants, the majority from TB-endemic countries. Lymph node TB (27/38 [71%]) was most frequent in EPTB. In the PTB group, 61% (41/70) were sputum PCR positive and 37% (26/70) had cavitary disease (Tables 1 and 2; Supplementary Figure 2; Supplementary Table 1). When TB was excluded (n = 204) by microbiology (98%) or clinical workup, non-TB respiratory tract infections were most common (76/204 [37%]), followed by chronic pulmonary diseases (34/204 [17%]) (Supplementary Table 3). To rule out TB disease, 86 of the participants had undergone 1 or more invasive diagnostic procedures.

**Table 1.** Characteristics of Study Participants

Characteristic	TB Disease	Non-TB Disease	Total	*P* Value
	n = 108	n = 204	n = 312	
Female sex	46 (42)	103 (50.5)	149 (47.8)	ns
Age, y, mean (range)	42 (18–89)	47 (19–89)	45 (18–89)	.026
Years since immigration, mean (range)	11. 4 (0.2 –71)	13.9 (0.2–63)	13 (0.2–71)	ns
Region (country of birth)				
Sub-Saharan Africa	33 (30.6)	65 (31.9)	98 (31.4)	ns
Europe	28 (25.9)	55 (27.0)	83 (26.6)	
Asia	35 (32.4)	48 (23.5)	83 (26.6)	
Middle East/North Africa	7 (6.5)	26 (12.7)	33 (10.6)	
Americas	5 (4.6)	10 (4.9)	15 (4.8)	
IGRA status				
Positive	84 (77.8)	108 (52.9)	192 (61.5)	<.001
Negative	5 (4.6)	81 (39.7)	86 (27.6)	
Indeterminate	6 (5.6)	4 (2.0)	10 (3.2)	
Not available	13 (12.0)	11 (5.4)	24 (7.7)	
Previous TB disease^a^	6 (5.6)	36 (17.6)	42 (13.5)	.003
Known TB infection^b^	15 (13.9)	29 (14.2)	44 (14.1)	ns
Immunosuppressing condition^c^, all	13 (12.0)	31 (15.2)	44 (14.1)	ns
Chronic lung disease	3 (2.8)	23 (11.3)	26 (8.3)	.010
Diabetes mellitus	6 (5.6)	15 (7.4)	21 (6.7)	ns

Data are presented as No. (%) unless otherwise indicated.

Abbreviations: IGRA, interferon-gamma release assay; ns, not significant; TB, tuberculosis.

^a^All had symptoms and/or radiological appearance suggesting possible recurrence of TB disease. Two individuals in the non-TB group were considered to have had a nonadequate TB treatment (12 and 43 years ago, respectively).

^b^Previously known and documented positive IGRA or purified protein derivative test (PPD).

^c^Human immunodeficiency virus (TB, n = 4; non-TB, n = 2); chronic kidney disease with glomerular filtration rate <60 ml/min/1.73m2 (TB, n = 3; non-TB, n = 5); hematological disease (TB, n = 1; non-TB, n = 7); rheumatological disease (TB, n = 3; non-TB, n = 16), with immunosuppressive therapy ongoing (n = 12) or within the last 6 months (n = 3); preexisting malignancy (TB, n = 3; non-TB, n = 4).

**Table 2.** Diagnoses of Study Participants

Characteristic	PTB	EPTB	All TB Disease	Non-TB Disease	*P* Value
	n = 70	n = 38	n = 108	n = 204	
Patient characteristics					
Female sex	26 (37)	20 (53)	46 (44)	103 (50)	ns
Age, y, mean (range)	42 (20–89)	42 (18–81)	42 (18–89)	47 (19–89)	PTB vs non TB: ns
					TB vs non TB: .026
General symptoms, mean (range)	1.8 (0–4)	0.8 (0–4)**	1.5 (0–4)	1.1 (0–4)*	PTB vs EPTB: .001
					TB vs non TB: .006
Fever (yes)	32 (46)	5 (13)	37 (34)	65 (32)	ns
Loss of appetite (yes)	27 (39)	5 (13)	32 (30)	36 (18)*	TB vs non TB: .015
Weight loss (yes)	39 (56)	12 (32)	51 (47)	57 (28)**	TB vs non TB: <.001
Night sweats (yes)	31 (44)	9 (24)	40 (37)	59 (29)	ns
Fatigue (yes)	33 (47)	10 (26)	43 (40)	45 (22)**	TB vs non TB: <.001
No. of general symptoms					
0	14 (20)	21 (55)	35 (32)	91 (45)*	TB vs non TB: .006
1	17 (24)	10 (26)	27 (25)	45 (22)*	
2	14 (20)	2 (5)	16 (15)	43 (21)*	
3	16 (23)	3 (8)	19 (18)	14 (7)*	
4	9 (13)	2 (5)	11 (10)	11 (5)*	
Cough (yes)	56 (80)	6 (16)	62 (57)	135 (66)	ns
Chest radiology					
Normal	0	18 (47)	18 (17)	70 (34)*	TB vs non TB: .011
Pathology CXR	44 (63)	5 (13)	49 (45)	70 (34)*	
Pathology CCT	26 (37)	13 (34)	39 (36)	60 (29)*	
Cavitation^a^	26 (37)	0	26 (24)	10 (5)	
Not done	0	2 (5)	2 (2)	4 (2)	
Basis for TB diagnosis					
*Mtb* culture	67 (96)	30 (79)	97 (90)	…	
*Mtb* PCR	2 (3)	3 (8)	5 (5)	…	
Histopathology	0	1 (3)	1 (1)	…	
Clinical	1 (1)	4^b^ (11)	5 (5)	…	
Diagnostic procedures performed					
Bronchoscopy	18 (26)	0	18 (17)	48 (26)	
Gastric lavage	13 (19)	0	13 (12)	20 (10)	
Biopsy	17 (24)	33 (87)	50 (46)	34 (17)	
Microscopy					
Sputum positive	29 (41)	0	29 (27)	0	
Other sample positive	4^c^ (6)	4^d^ (11)	8 (7)	0	
*Mtb* PCR (BD MAX)					
Sputum positive	43 (61)	0	43 (13)	0	
Other airway sample positive	7^e^ (10)	0	7 (6)	0	
Other sample positive	6 (9)	23 (61)	29 (27)	0	
*Mtb* culture, any					
Positive	67 (96)	30 (79)	97 (90)	0	
Negative	3 (4)	5 (13)	8 (7)	199 (98)	
Not performed	0	3^f^ (8)	3 (3)	5^g^ (2)	
Sputum *Mtb* culture					
Positive	52 (74)	0	52 (48)	0	
Negative	12 (17)	16 (42)	28 (26)	184 (90)	
Not performed	6 (9)	22 (58)	28 (26)	20 (10)	
Nonsputum *Mtb* culture					
Positive	43^h^ (61)	30 (79)	73 (68)	0	
Negative	1 (1)	5 (13)	6 (6)	95 (47)	
Not performed	26 (37)	3 (8)	3 (3)	109 (53)	
MTB-HR, TBP score^i^					
Capillary, mean (SD)	−2.15 (1.002)	−1.82 (0.689)	−2.040 (0.911)	−0.975 (0.583)	TB vs non TB: <.001
					PTB vs EPTB: ns
Venous, mean (SD)	−2.15 (0.989)	−1.93 (0.744)	−2.077 (0.924)	−0.981 (0.640)	TB vs non TB: <.001
					PTB vs EPTB: ns

Data are presented as No. (%) unless otherwise indicated. Mann-Whitney test comparing means. Statistical comparison between PTB and EPTB and TB disease to non-TB disease: *P < .05, **P < .001. Pearson χ^2^ test to compare proportions.

Abbreviations: CCT, chest computed tomography; CXR, chest radiography; EPTB, extrapulmonary tuberculosis; Mtb, *Mycobacterium tuberculosis*; MTB-HR, Xpert MTB Host Response; ns, not significant; PCR, polymerase chain reaction; PTB, pulmonary tuberculosis; SD, standard deviation; TB, tuberculosis.

^a^Detected by CXR (n = 9) and CCT (n = 17) in PTB; CXR (n = 5) and CCT (n = 5) in non-TB; for example, lymphadenopathy or signs of past TB such as calcifications or scarring with volume loss.

^b^Not included in receiver operating characteristic analysis.

^c^Two bronchoalveolar lavage (BAL), two gastric lavage (GL).

^d^Two lymph node aspirates, two abscess aspirates.

^e^Four BAL, three GL.

^f^Three individuals with ocular TB.

^g^Four individuals in whom symptoms resolved, one individual lost to follow-up.

^h^Sixteen individuals only positive in nonsputum samples.

^i^MTB-HR TBP score = (Ct^GBP5^ + Ct^DUSP3^) / 2 – Ct^TBP^.

### Diagnostic Performance of MTB-HR in PTB and EPTB

**Figure 2.**
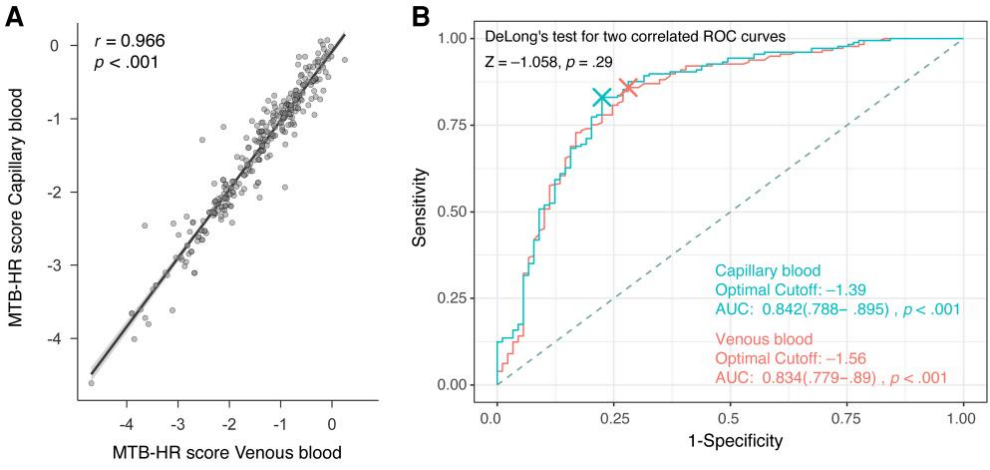
*A*, Pearson correlation between Xpert MTB Host Response (MTB-HR) TBP score in venous and capillary samples. Paired venous and capillary samples at study inclusion (n = 285). *B*, Receiver operating characteristic (ROC) curves generated based on capillary (n = 273) and venous (n = 289) samples. No significant difference between ROC curves, DeLong test (*P* = .29). Areas under the curve (AUCs) are shown with 95% confidence intervals. MTB-HR TBP score = (Ct^GBP5^ + Ct^DUSP3^) / 2 – Ct^TBP^.

**Figure 3.**
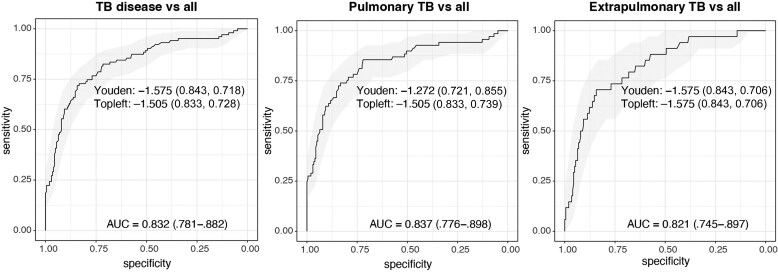
Xpert MTB Host Response (MTB-HR) performance in confirmed tuberculosis (TB) disease. Receiver operating characteristic curves for all TB disease (n = 103, left), pulmonary TB (n = 69, middle), and extrapulmonary TB (n = 34, right); areas under the curve (AUC) with shaded areas corresponding to the 95% confidence interval; Youden index and closest Topleft criterion test cut-offs with corresponding specificity and sensitivity in parentheses. Pooled venous and capillary patient samples were used in the analysis. MTB-HR score = (Ct^GBP5^ + Ct^DUSP3^) / 2 – Ct^TBP^.

**Figure 4.**
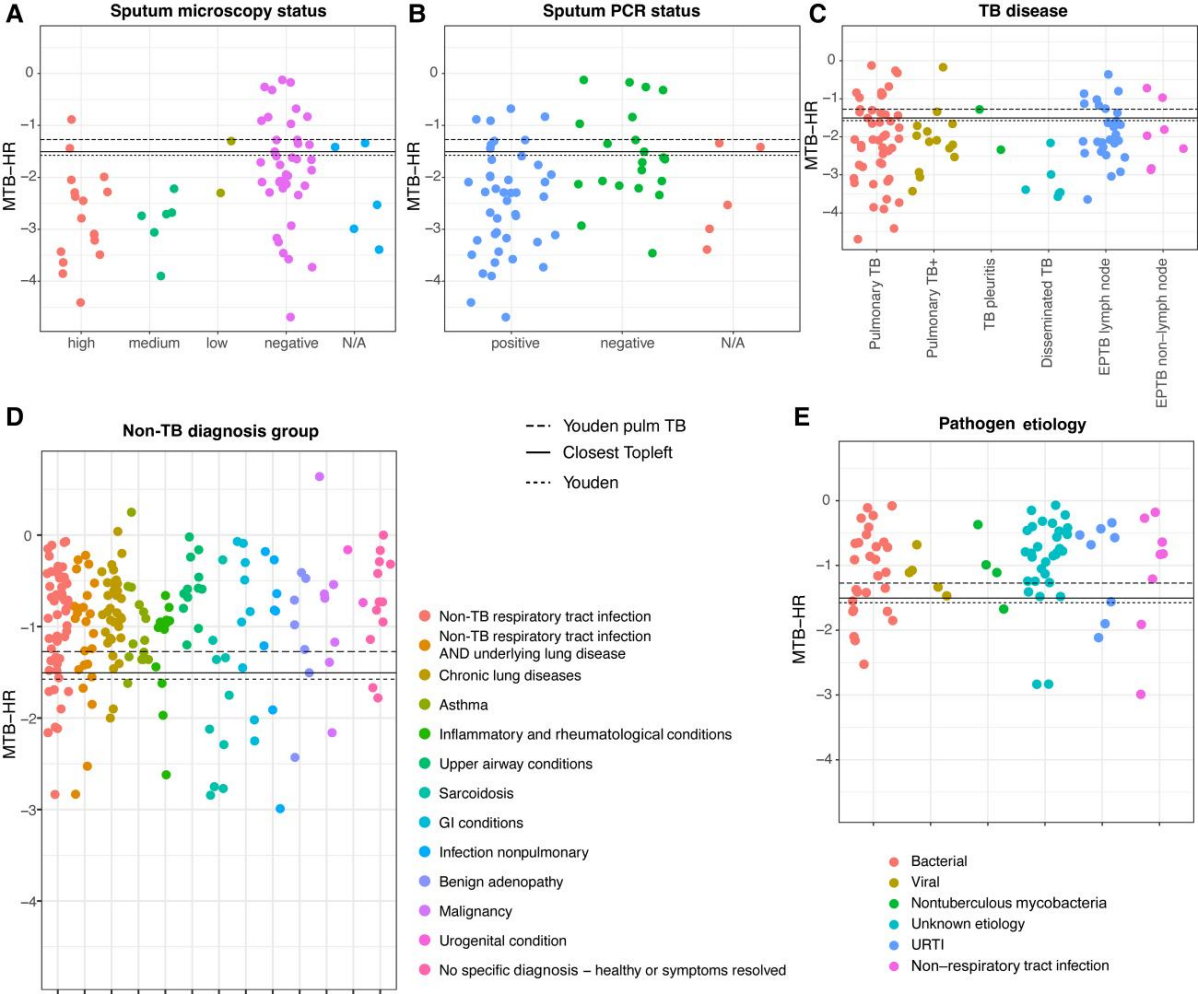
Xpert MTB Host Response (MTB-HR) score according to microbiological results in pulmonary tuberculosis (TB) and different subgroups of TB and non-TB disease. For all panels, the dashed lines represent the overall Youden index cut-off −1.58, the black line the overall closest Topleft cut-off −1.51, and the dotted line the Youden index cut-off −1.27 for pulmonary TB. A value below the cut-off indicates a positive MTB-HR result for TB disease. *A*, MTB-HR in pulmonary TB, grouped by sputum microscopy (high, medium, low/negative, not available). *B*, Sputum polymerase chain reaction (PCR) results (positive, negative, not available). The most positive sputum result is used for each individual. Five individuals could not produce a sputum sample. Twenty-six of 35 microscopy-negative and 15 of 21 PCR-negative individuals were identified using the pulmonary TB Youden cut-off −1.27, as were the 5 non-sputum-producing individuals. One sputum microscopy-positive and 4 PCR-positive individuals had a value above the cut-off. *C*, MTB-HR in different TB subgroups. “Pulmonary TB+” indicates an involvement of lymph nodes or pleura in addition to the lung parenchyma, and all cases of disseminated TB (affecting ≥2 organs) had lung involvement. In disseminated TB, all scores were below either cut-off. In non–lymph node extrapulmonary TB, the 2 individuals above the cut-offs were 1 patient with TB otitis and 1 patient with abdominal/gynecological TB. *D*, MTB-HR in non-TB disease by final diagnosis group. *E*, MTB-HR in non-TB respiratory tract infections, grouped by pathogen etiology, and in non–respiratory tract infections. Pooled venous and capillary patient samples were used in the analysis. MTB-HR score = (Ct^GBP5^ + Ct^DUSP3^) / 2 – Ct^TBP^. Abbreviations: EPTB, extrapulmonary tuberculosis; GI, gastrointestinal; MTB-HR, Xpert MTB Host Response; N/A, not available; PCR, polymerase chain reaction; TB, tuberculosis; URTI, upper respiratory tract infection.

Venous and capillary scores in paired samples were highly correlated (*r* = 0.97, *P* < .001, n = 285), with equal performance (Figure 2). The AUC for MTB-HR in TB disease was 0.83 (95% CI, .78–.88), similar for PTB (0.84 [95% CI, .78–.90]) and EPTB (0.82 [95% CI, .75–.90]), compared to a complete diagnostic workup. The Youden index cut-off was −1.27 for PTB and −1.58 for EPTB (Figure 3). The closest Topleft criterion resulted in a different cut-off for PTB of −1.51, yielding a higher specificity but lower sensitivity. In culture-confirmed PTB, an additional 26 of 35 microscopy-negative and 15 of 21 PCR-negative individuals were identified using the PTB Youden cut-off, as were the 5 non-sputum-producing individuals (Figure 4*A* and 4*B*).

### MTB-HR Score in Relation to Etiology, Bacillary Burden, and WHO TPP Requirements

The MTB-HR score ranged from −4.69 to 0.64, with a more negative value indicating a stronger signal; treatment initiation up to 3 days earlier (16 individuals) did not significantly affect the MTB-HR score. The mean score was −2.10 (95% CI, −2.28 to −1.91) in TB disease overall, with the highest score in disseminated TB involving 2 or more organs (−3.18 [95% CI, −3.74 to −2.61]), and −0.99 (95% CI, −1.08 to −.90) in individuals with other diagnoses (Figure 4*C–E*; Supplementary Tables 2 and 3). The signature had a poor performance in sarcoidosis (mean score, −1.86) whereas there was no clear trend with regard to etiology of non-TB infections.

**Figure 5.**
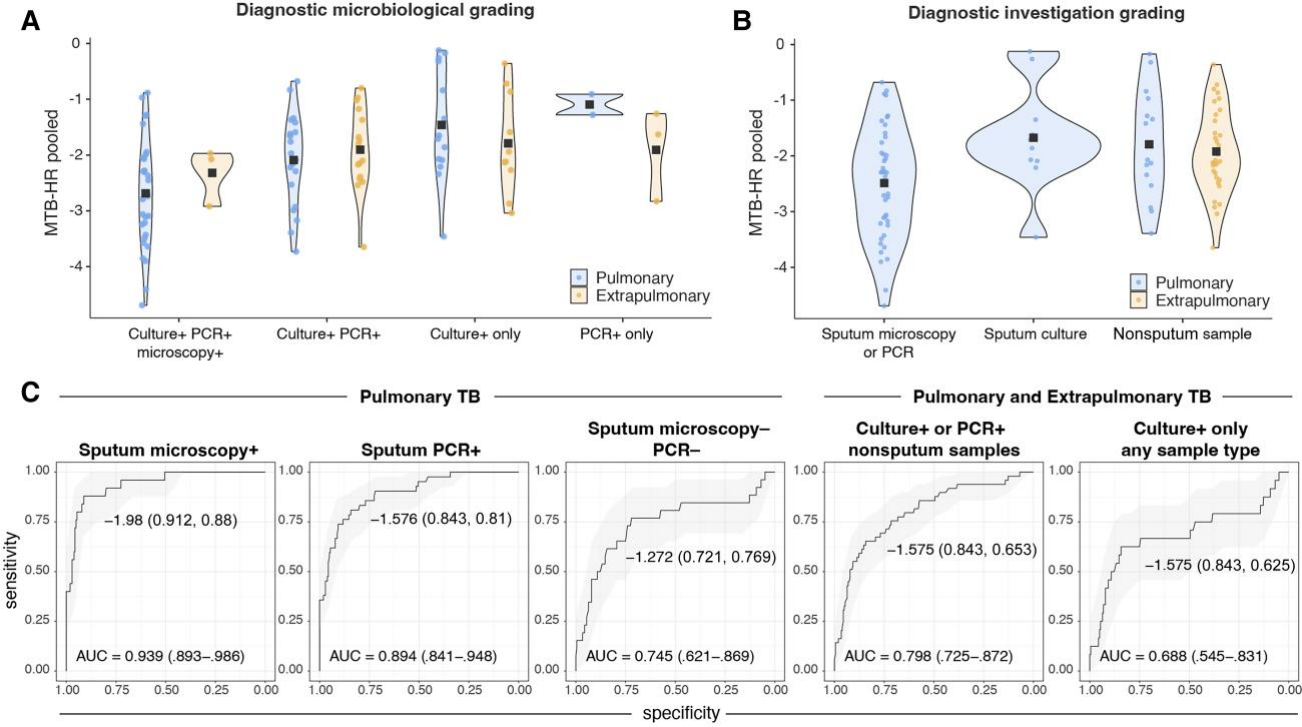
Xpert MTB Host Response (MTB-HR) score in pulmonary and extrapulmonary tuberculosis (TB) grouped by (*A*) microbiological grading: culture positive, polymerase chain reaction (PCR) positive, microscopy positive; culture positive, PCR positive; culture positive, PCR negative; and culture negative, PCR positive; and (*B*) diagnostic grading: sputum microscopy positive and/or PCR positive; sputum sample (microscopy negative and PCR negative); and nonsputum sample. MTB-HR score = (Ct^GBP5^ + Ct^DUSP3^) / 2 – Ct^TBP^. *C*, Receiver operating characteristic (ROC) analysis of MTB-HR score diagnostic performance in microbiologically confirmed TB, grouped by diagnostic characteristics. From left to right: In pulmonary TB: microscopy positive, PCR positive, culture positive; PCR positive, culture positive; microscopy negative, PCR negative, culture positive. In extrapulmonary TB and pulmonary TB combined: culture positive or PCR positive in nonsputum sample; culture positive only in sputum or nonsputum samples. Cut-offs based on Youden index; specificity and sensitivity are shown in parentheses; the shaded area shows the 95% confidence interval. Pooled venous and capillary patient samples were used in the analysis.

In confirmed PTB and EPTB, the mean MTB-HR score was significantly stronger in microscopy-positive, PCR-positive, culture-positive (−2.61 [95% CI, −2.96 to −2.34]; n = 35), compared to PCR-positive, culture-positive (−2.01 [95% CI, −2.27 to −1.750]; n = 37; *P =* .013) or culture-positive only (−1.60 [95% CI, −2.00 to −1.196]; n = 24; *P <* .001) (Figure 5*A* and 5*B*). Subgroup ROC analysis produced matching results (Figure 5*C*), with highest performance in sputum microscopy-positive (AUC, 0.94; sensitivity 88%, specificity 91%) and slightly lower in sputum PCR-positive (AUC, 0.90; sensitivity 81%, specificity 84%) PTB. In culture-positive only paucibacillary PTB, AUC was 0.75 (sensitivity 77%, specificity 72%). When invasive procedures were necessary for microbiological confirmation in both EPTB and PTB, AUC was 0.79 (sensitivity 65%, specificity 84%).

The TTP in sputum correlated moderately to MTB-HR (*r* = 0.463, *P* < .001; Supplementary Figure 3). TB infection defined by positive interferon-gamma release assay (IGRA) status did not impact test results, with no significant differences in mean MTB-HR score between IGRA-positive and IGRA-negative individuals in either study group (Supplementary Figure 3).

Benchmarking against the WHO TPP (Supplementary Table 4), the 90% sensitivity target was reached in microscopy-positive PTB (cut-off −1.43, specificity 81%, NPV 98.5%), whereas in PCR-positive PTB, the specificity was 71% (cut-off −1.27, NPV 97.2%). Applying the suggested cut-off −1.25 [19], MTB-HR achieved 86% sensitivity and 72% specificity with NPV 94% for PTB overall. For EPTB, MTB-HR exceeded the minimum 65% sensitivity requirement for culture-confirmed TB, yielding a high NPV of 95% (cut-off −1.58, sensitivity 71%, specificity 84%).

### Analysis of MTB-HR in Relation to Demographic Factors, Clinical Symptoms, and Inflammatory Markers

Performance was best in the age group <25 years (AUC, 0.97), with similar performance in the older age spans, and higher in men compared to women, with the sex difference being more pronounced in EPTB (AUC, 0.88 in men vs 0.75 in women). In individuals with immunosuppressive conditions (n = 44), MTB-HR performed equally well as in non-immunosuppressed individuals (AUC, 0.87 vs 0.83) for TB disease overall (Supplementary Figure 4). Clinical parameters (CRP and ESR, number of typical TB symptoms) showed a stronger correlation to MTB-HR in individuals with TB disease (Supplementary Figures 5 and 6). Routine biochemical markers were less discriminative of TB disease than MTB-HR, with CRP having the highest AUC of 0.70 with a cut-off of 8 mg/L (Supplementary Figure 7).

## DISCUSSION

In this prospective study in a TB low-endemic, high-resource setting, Xpert MTB-HR had a high performance in confirmed PTB and EPTB (AUC, 0.82–0.84). MTB-HR improves early detection of PCR-negative PTB, including disseminated TB, and is promising as a rule-out test in EPTB (NPV 95%). In our cohort, the Youden index cut-off was −1.27 for PTB, compared to the suggested cut-off −1.25 [19], both with an 86% sensitivity and 72% versus 71% specificity close to the WHO TPP target, and an NPV of 94% for PTB.

In EPTB, TPP requirements are not as clearly defined as in PTB; however, MTB-HR (cut-off −1.58) reached the 2014 overall diagnostic sensitivity test target with 71% sensitivity (84% specificity). In the 2024 updated TPP [13], the target for a nonsputum near-POC test is set at 75% sensitivity for culture-confirmed TB, without separate thresholds for EPTB. To our knowledge, no previous evaluation of the Xpert MTB-HR cartridge in EPTB has been published. Two studies, Hoang et al [25] and a preprint by Mann et al [26], have evaluated transcriptional signatures in EPTB cohorts [25, 26], the latter focusing exclusively on lymph node TB and TB pericarditis. In these studies, Sweeney3 AUC was 0.83 and 0.76, respectively, outperforming CRP in comparison [26]. However, these studies did not evaluate the TBP score nor the Xpert MTB-HR cartridge and had a lower rate of microbiological confirmation, affecting overall validity.

In a direct comparison, performance of venous and capillary MTB-HR sampling was equal, facilitating screening and near-POC testing. In pediatric TB, with its well-known diagnostic challenges [27, 28], capillary testing is preferred. In a first study including children aged <15 years, Xpert MTB-HR in finger-stick blood had a sensitivity of 60% with 90% specificity for culture-confirmed TB [29]. The cartridge with the TBP score also performed very well in the age group 10–24 years, as shown recently by Seifert et al [30], consistent with our results in the youngest adult age group (<25 years). Aging may impact TB-specific immunity as previously described [31], but our material did not allow further analysis of this aspect.

Xpert MTB-HR evaluations in low- and medium-resource settings have reported higher AUCs of 0.88 and 0.89 for PTB [18, 19] and improved performance when the reference was Xpert MTB Ultra rather than *Mtb* culture [18]. Additionally, accuracy was higher in patients with higher bacterial load as indicated by Xpert MTB/RIF [17, 32]. We believe the enhanced performance in these studies could be attributed to the exclusion of paucibacillary disease due to limited access to invasive diagnostic procedures where sputum PCR-negative and non-sputum-producing individuals may remain undetected. We found that in TB diagnosis based on nonsputum samples and culture only, sensitivity was reduced (65% and 63%, respectively) while specificity remained high (84%). We also identified a correlation between MTB-HR and MGIT TTP in sputum and significantly stronger scores in microscopy-positive and PCR-positive TB, supporting this hypothesis. Nonetheless, with a standard of 3 sputum samples per patient in our cohort, Xpert MTB-HR sensitivity (86%) using the PTB Youden index cut-off −1.27 was still superior to sputum microscopy (41%) and *Mtb* PCR (61%), making it useful for early diagnosis and detection of paucibacillary TB.

### Clinical Implications

We propose Xpert MTB-HR as a diagnostic tool in EPTB with a sensitivity of 71% and high specificity of 84% (NPV 95%), offering guidance as to whether invasive TB sampling is warranted. Although there are few observations, our results indicate that MTB-HR has a stronger signal in more severe, clinically challenging forms of EPTB such as abdominal and musculoskeletal TB. In settings where invasive procedures for microbiological sampling are unfeasible, or patient condition too critical to await culture results, the result of Xpert MTB-HR could also support initiation of empiric TB treatment.

In PTB, it has been suggested that transcriptomic signatures are important to identify individuals with difficult-to-diagnose PTB with negative sputum microscopy and PCR [33]. Indeed, we found that MTB-HR contributed to diagnosis of patients unable to produce sputum and paucibacillary PTB verified only by *Mtb* culture, with a higher sensitivity than sputum PCR and an NPV of 94%. Microbiological testing is necessary to evaluate contagiousness and antimicrobial resistance; however, Xpert MTB-HR could be used as a complementary nonsputum rule-out test, given its quick response time and the ease of capillary testing.

Moreover, our results imply that MTB-HR may be useful in disseminated TB where all patients were identified by MTB-HR, but only 2 of 7 patients had a positive sputum microscopy or PCR result despite microbiologically verified lung involvement. Optimal cut-offs for PTB and EPTB differed, and MTB-HR was strongly correlated to bacterial burden and disease severity in TB disease, thus suggesting the added value of a graded score in accordance with other commonly used biomarkers. A severely ill patient with an MTB-HR score above cut-off would suggest that other diagnoses are more likely. With negative scores being counterintuitive and possibly difficult to interpret for clinicians, transforming the score to an absolute value or using a graded score combined with diagnostic algorithms for different clinical situations could be envisaged. Exact cut-offs would need to be confirmed in larger patient cohorts, but we want to emphasize that a binary cut-off would be too simplified, with different cut-offs relevant depending on the type of patient.

### Strengths

This is the first study to evaluate the Xpert MTB-HR prototype cartridge in EPTB, which, in endemic settings, mostly lacks microbiological confirmation. The major strength of our study is the high level of diagnostic microbiological and histopathological confirmation in EPTB and PTB alike, including individuals with sputum PCR-negative and disseminated TB. Conducted in a high-resource setting with a thoroughly investigated cohort with a very low risk of undiagnosed TB, we have scrutinized MTB-HR performance compared to a full diagnostic workup. The TB cohort composition is comparable to TB high-endemic contexts, and the diverse geographical patient background enhances generalizability to other settings.

### Limitations

We have examined the Xpert MTB-HR results in different types of TB disease; however, clinical subgroups, such as disseminated TB, are too small to draw strong conclusions or define separate cut-offs. The gender difference in diagnostic performance seen in EPTB also needs confirmation in larger patient cohorts. HIV prevalence in our setting is low; nonetheless, test performance in immunosuppressed individuals was not reduced. Study inclusion was done in routine care during the coronavirus disease 2019 pandemic, which meant that fewer patients were seen at the clinic than anticipated. Even so, we consider the study population to be representative of our regular patient population. In the Stockholm region there were 322 reported cases of TB disease during the years 2020–2022.

## CONCLUSIONS

Xpert MTB-HR was particularly useful in difficult-to-diagnose individuals, such as those who cannot produce sputum, paucibacillary PTB only detected by culture, and EPTB. It could complement existing microbiological methods by being more sensitive than sputum PCR, quicker than *Mtb* culture, and with MTB-HR score closely linked to bacterial burden. In EPTB, the high NPV of MTB-HR can be used as a rule-out test, while it also shows potential as an add-on diagnostic tool to be explored in the development of diagnostic algorithms. We suggest a graded MTB-HR score as more clinically relevant than a binary test result.

## Supplementary Material

jiaf110_Supplementary_Data

## Supplementary Data

Supplementary materials are available at *The Journal of Infectious Diseases* online (http://jid.oxfordjournals.org/). Supplementary materials consist of data provided by the author that are published to benefit the reader. The posted materials are not copyedited. The contents of all supplementary data are the sole responsibility of the authors. Questions or messages regarding errors should be addressed to the author.

## Notes

***Acknowledgments.*** The collaboration of all study participants made this study possible. The TB nurses and physicians at the Department of Infectious Diseases, Karolinska University, were instrumental in the recruitment and follow-up of study participants. Zainab Mousavian provided valuable methodological input. Statistical analysis was conducted with support from the Biostatistics Core Facility, Karolinska Institutet.

***Author contributions.*** E. F., G. F., and J. B., with input from E. S., conceptualized and designed the study. E. F., G. F., and J. B. conducted and coordinated the research activity. C. S. provided scientific support. E. S. provided technical support. E. F. and J. B. did the formal data analysis. E. F. wrote the manuscript draft. All authors had substantial input in the preparation of the manuscript. E. F., G. F., and J. B. had access to and verified all the data. All authors contributed to the interpretation of data, reviewed and edited the manuscript, approved the final version of the manuscript, and had final responsibility for the decision to submit the manuscript for publication.

***Data sharing.*** Anonymized individual participant data that underlie the results reported in this article can be shared with investigators for prespecified purposes, such as meta-analysis, after individual consideration. Data sharing on condition of approval by an independent ethical review board and pending complimentary ethical permits being obtained from the Swedish Ethical Review Board.

***Disclaimer.*** Cepheid contributed to study planning and data collection but did not influence data analysis or conclusions.

***Financial support.*** Funding from Cepheid supported this work, contributing to salary costs of study personnel. Cepheid also supplied cartridges and GeneXpert machines. The Swedish Heart Lung Foundation (grant number 20230511) also supported this work.

## References

[1] World Health Organization (WHO) . End TB strategy. Geneva, Switzerland, WHO, 2015.

[2] Pai M, Dewan PK, Swaminathan S. Transforming tuberculosis diagnosis. Nat Microbiol 2023; 8:756–9. 37127703 10.1038/s41564-023-01365-3

[3] Subbaraman R, Nathavitharana RR, Mayer KH, et al Constructing care cascades for active tuberculosis: a strategy for program monitoring and identifying gaps in quality of care. PLoS Med 2019; 16:e1002754. 30811385 10.1371/journal.pmed.1002754 PMC6392267

[4] World Health Organization (WHO) . Global tuberculosis report 2024. Geneva, Switzerland: WHO, 2024.

[5] Getnet F, Demissie M, Assefa N, Mengistie B, Worku A. Delay in diagnosis of pulmonary tuberculosis in low-and middle-income settings: systematic review and meta-analysis. BMC Pulm Med 2017; 17:202. 29237451 10.1186/s12890-017-0551-y PMC5729407

[6] Norbis L, Alagna R, Tortoli E, Codecasa LR, Migliori GB, Cirillo DM. Challenges and perspectives in the diagnosis of extrapulmonary tuberculosis. Expert Rev Anti Infect Ther 2014; 12:633–47. 24717112 10.1586/14787210.2014.899900

[7] Kohli M, Schiller I, Dendukuri N, et al Xpert MTB/RIF Ultra and Xpert MTB/RIF assays for extrapulmonary tuberculosis and rifampicin resistance in adults. Cochrane Database Syst Rev 2021; 1:CD012768. 33448348 10.1002/14651858.CD012768.pub3 PMC8078545

[8] Sharma SK, Mohan A, Kohli M. Extrapulmonary tuberculosis. Expert Rev Respir 2021; 15:931–48. 10.1080/17476348.2021.1927718 33966561

[9] Gong X, He Y, Zhou K, Hua Y, Li Y. Efficacy of Xpert in tuberculosis diagnosis based on various specimens: a systematic review and meta-analysis. Front Cell Infect Microbiol 2023; 13:1149741. 37201118 10.3389/fcimb.2023.1149741 PMC10185844

[10] Jain R, Gupta G, Mitra DK, Guleria R. Diagnosis of extrapulmonary tuberculosis: an update on novel diagnostic approaches. Respir Med 2024; 225:107601. 38513873 10.1016/j.rmed.2024.107601

[11] European Centre for Disease Prevention and Control; World Health Organization Regional Office for Europe . Tuberculosis surveillance and monitoring in Europe 2022–2020 data. Copenhagen and Stockholm: WHO Regional Office for Europe/European Centre for Disease Prevention and Control, 2022.

[12] World Health Organization (WHO) . High priority target product profiles for new tuberculosis diagnostics: report of a consensus meeting, 28–29 April 2014. Geneva, Switzerland: WHO, 2014.

[13] World Health Organization (WHO) . Target product profile for tuberculosis diagnosis and detection of drug resistance. Geneva, Switzerland: WHO, 2024.

[14] Sweeney TE, Braviak L, Tato CM, Khatri P. Genome-wide expression for diagnosis of pulmonary tuberculosis: a multicohort analysis. Lancet Respir Med 2016; 4:213–24. 26907218 10.1016/S2213-2600(16)00048-5 PMC4838193

[15] Warsinske HC, Rao AM, Moreira FMF, et al Assessment of validity of a blood-based 3-gene signature score for progression and diagnosis of tuberculosis, disease severity, and treatment response. JAMA Netw Open 2018; 1:e183779. 30646264 10.1001/jamanetworkopen.2018.3779 PMC6324428

[16] Södersten E, Ongarello S, Mantsoki A, et al Diagnostic accuracy study of a novel blood-based assay for identification of tuberculosis in people living with HIV. J Clin Microbiol 2021; 59:e01643-20. 33298607 10.1128/JCM.01643-20 PMC8106701

[17] Moreira FMF, Verma R, Pereira Dos Santos PC, et al Blood-based host biomarker diagnostics in active case finding for pulmonary tuberculosis: a diagnostic case-control study. EClinicalMedicine 2021; 33:100776. 33842866 10.1016/j.eclinm.2021.100776 PMC8020164

[18] Sutherland JS, van der Spuy G, Gindeh A, et al Diagnostic accuracy of the Cepheid 3-gene host response fingerstick blood test in a prospective, multi-site study: interim results. Clin Infect Dis 2022; 74:2136–41. 34550342 10.1093/cid/ciab839 PMC9258935

[19] Gupta-Wright A, Ha H, Abdulgadar S, et al Evaluation of the Xpert MTB Host Response assay for the triage of patients with presumed pulmonary tuberculosis: a prospective diagnostic accuracy study in Viet Nam, India, the Philippines, Uganda, and South Africa. Lancet Glob Health 2024; 12:e226–34. 38245113 10.1016/S2214-109X(23)00541-7 PMC11046618

[20] Levy H, Feldman C, Sacho H, van der Meulen H, Kallenbach J, Koornhof H. A reevaluation of sputum microscopy and culture in the diagnosis of pulmonary tuberculosis. Chest 1989; 95:1193–7. 2656111 10.1378/chest.95.6.1193

[21] DeLong ER, DeLong DM, Clarke-Pearson DL. Comparing the areas under two or more correlated receiver operating characteristic curves: a nonparametric approach. Biometrics 1988; 44:837–45. 3203132

[22] Martínez-Camblor P, Pardo-Fernández JC. The Youden index in the generalized receiver operating characteristic curve context. Int J Biostat 2019; 15:20180060. 10.1515/ijb-2018-0060 30943172

[23] Perkins NJ, Schisterman EF. The inconsistency of “optimal” cutpoints obtained using two criteria based on the receiver operating characteristic curve. Am J Epidemiol 2006; 163:670–5. 16410346 10.1093/aje/kwj063 PMC1444894

[24] Jamovi Project . jamovi. Version 2.4. 2023. https://www.jamovi.org. Accessed January 2024.

[25] Hoang LT, Jain P, Pillay TD, et al Transcriptomic signatures for diagnosing tuberculosis in clinical practice: a prospective, multicentre cohort study. Lancet Infect Dis 2021; 21:366–75. 33508221 10.1016/S1473-3099(20)30928-2 PMC7907671

[26] Mann T, Minnies S, Gupta RK, et al Blood RNA signatures outperform CRP triage of tuberculosis lymphadenitis and pericarditis. medRxiv [Preprint]. Posted online 3 July 2024. 10.1101/2024.06.21.2430909.

[27] Thomas TA . Tuberculosis in children. Pediatr Clin North Am 2017; 64:893–909. 28734517 10.1016/j.pcl.2017.03.010 PMC5555046

[28] Detjen AK, DiNardo AR, Leyden J, et al Xpert MTB/RIF assay for the diagnosis of pulmonary tuberculosis in children: a systematic review and meta-analysis. Lancet Respir Med 2015; 3:451–61. 25812968 10.1016/S2213-2600(15)00095-8 PMC4756280

[29] Olbrich L, Verghese VP, Franckling-Smith Z, et al Diagnostic accuracy of a three-gene *Mycobacterium tuberculosis* Host Response cartridge using fingerstick blood for childhood tuberculosis: a multicentre prospective study in low-income and middle-income countries. Lancet Infect Dis 2024; 24:140–9. 37918414 10.1016/S1473-3099(23)00491-7 PMC10808504

[30] Seifert M, Catanzaro DG, Gracia M, et al Prospective exploratory evaluation of Cepheid Xpert *Mycobacterium tuberculosis* Host Response cartridge: a focus on adolescents and young adults. Clin Infect Dis 2025; 80:180–8. 39233548 10.1093/cid/ciae461 PMC11797382

[31] Grifoni A, Alonzi T, Alter G, et al Impact of aging on immunity in the context of COVID-19, HIV, and tuberculosis. Front Immunol 2023; 14:1146704. 37292210 10.3389/fimmu.2023.1146704 PMC10246744

[32] Li M, Qiu Y, Guo M, et al Evaluation of the Cepheid 3-gene host response blood test for tuberculosis diagnosis and treatment response monitoring in a primary-level clinic in rural China. J Clin Microbiol 2023; 61:e0091123. 37902328 10.1128/jcm.00911-23 PMC10662368

[33] Greenan-Barrett J, Gupta RK, Noursadeghi M. The end of the road for blood RNA biomarkers as triage tests for symptomatic pulmonary tuberculosis among spontaneous sputum producers? Eur Resp J 2024; 64:2401365. 10.1183/13993003.01365-2024 39147423

