# Supplementary material for: Improved Detection of Extrapulmonary and Paucibacillary Pulmonary Tuberculosis by Xpert MTB Host Response in a Tuberculosis Low-Endemic, High-Resource Setting
Source: J Infect Dis. 2025 Mar 6;232(1):e78–88. doi: 10.1093/infdis/jiaf110 (PMC12308647; doi:10.1093/infdis/jiaf110)
Supplement: jiaf110_Supplementary_Data [file jiaf110_supplementary_data.zip › Folkesson_Supplement_Figuresandtables_rev.pdf]

Supplemental figures and tables

Improved detection of extrapulmonary and paucibacillary pulmonary tuberculosis by Xpert MTB Host Response in a TB low endemic, high resource setting

Figure S1. ROC-analysis of MTB-HR TBP-score and KLF2-score page 2

Figure S2. Types and number of cases of TB disease. page 3

Figure S3. MTB-HR score by IGRA-status in non-TB and TB disease. page 3

Figure S4. MTB-HR ROC-curves according to age, sex, immunosuppression and country of birth. page 4

Figure S5 Correlation MTB-HR score to MGIT time to positive growth, ESR and CRP. page 5

Figure S6. MTB HR TBP in relation to number of B-symptoms in TB and non-TB disease. page 5

Figure S7. ROC-analysis of blood biomarkers for TB disease page 6

Table S1. Biochemistry results in Pulmonary TB, extrapulmonary TB and non-TB disease. page 7

Table S2. MTB-HR, TBP score and type of samples. page 8

Table S3. Non-TB disease cohort final diagnosis page 9

Table S4. Xpert MTB-HR test performance in TB disease page 10

Supplemental Figures

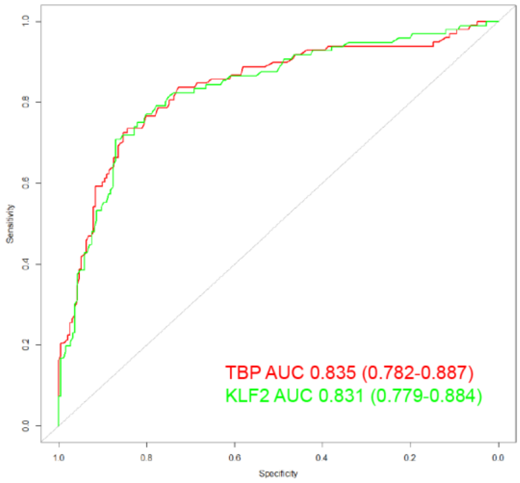

Figure S1. ROC-curves for MTB-HR; TBP-based score (AUC 0.835) and KLF2-based score (AUC 0.831). TBP-score: (CtGBP5+CtDUSP3)/2 – CtTBP. KLF2-score: (CtGBP5+CtDUSP3)/2 – CtKLF2

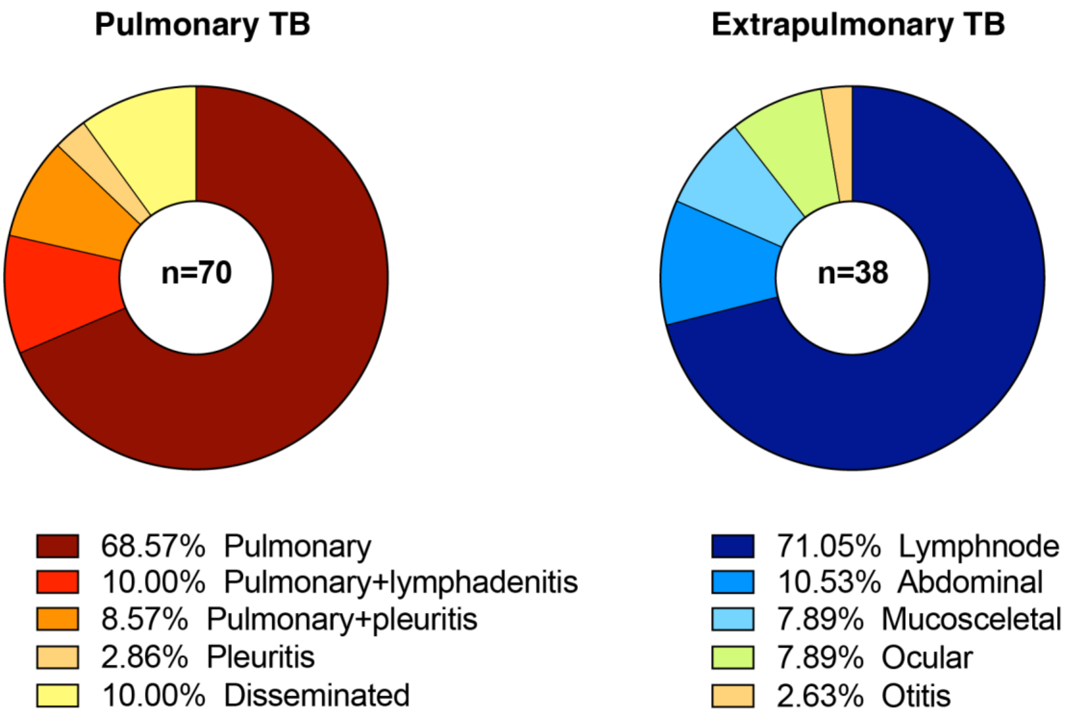

Figure S2. Types and number of cases of TB disease in; Pulmonary TB including disseminated disease (left panel, n=70) and Extrapulmonary TB (right panel, n=38). Disseminated TB defined as involvement in $\geq$ 2 organs, not including pulmonary TB with thoracic lymphadenopathy. Four individuals with EPTB were excluded from statistical analysis (3 ocular TB, 1 abdominal TB)

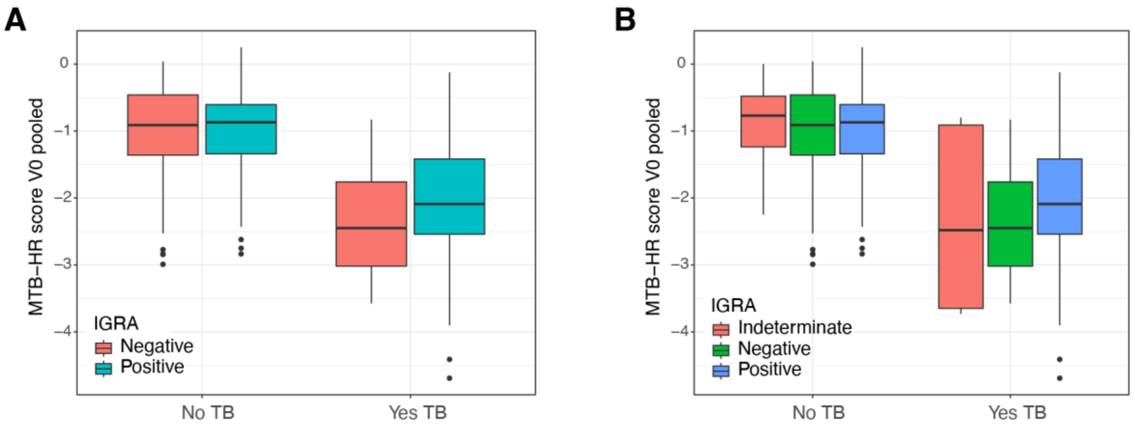

Figure S3. MTB-HR score by IGRA-status in non-TB and TB disease. There was no significant difference within either group between **A)** IGRA+ and IGRA– ;TB disease p = 0.686, non-TB: 0.868. **B)** IGRA+, IGRA – and IGRA indeterminate. TB disease: p=0.947; non TB:p= 0.926. One-way ANOVA used for comparison

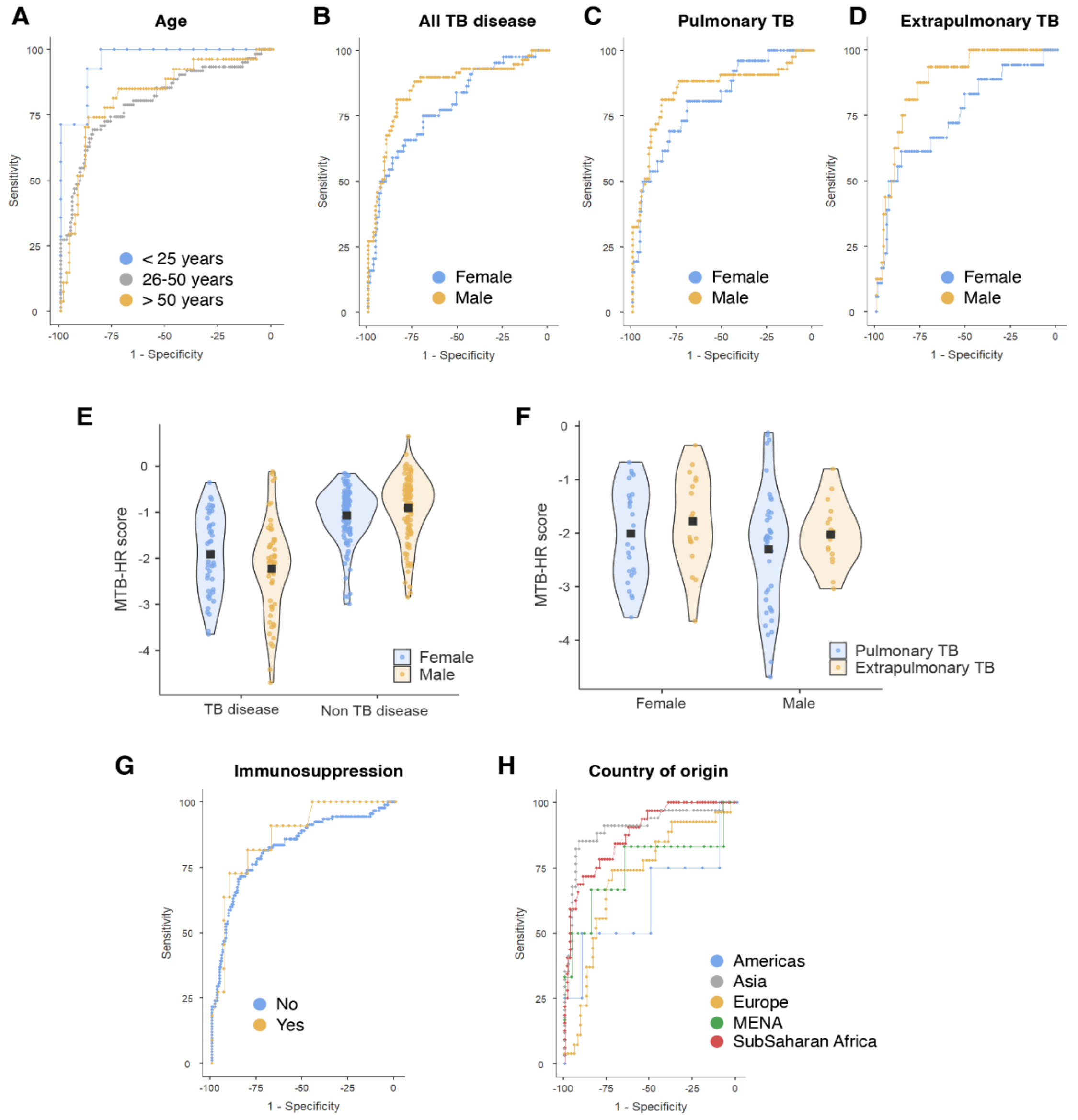

Figure S4. A) ROC curves for MTB-HR by age group: 1: ≤25 y. AUC 0.96 2. 26-50: AUC 0.809 3. ≥50: AUC 0.828. **B)** ROC curves for MTB-HR by sex, all TB: Female: AUC 0.787 Male: AUC 0.857. **C)** in PTB Female: AUC 0.811. Male: AUC 0.849. **D)** in EPTB Female: AUC 0.752. Male: AUC 0.880. **E)** MTB-HR by sex in TB disease and non TB. **F)** MTB-HR in PTB and EPTB. Females and males. Mean MTB HR score more negative in males than females, no statistically significant difference by Mann-Whitney U test in neither comparison. **G)** ROC curves for MTB-HR in immunosuppression: AUC 0.871 and no immunosuppression: AUC 0.829. **H)** ROC curves by country of origin: Americas: n =15, AUC 0.625. Asia: n=83, AUC 0.907. Europe: n=83, AUC 0.720. MENA: n=33, AUC 0.756. Sub Saharan Africa: n=98, AUC 0.883. MENA = Middle East North Africa

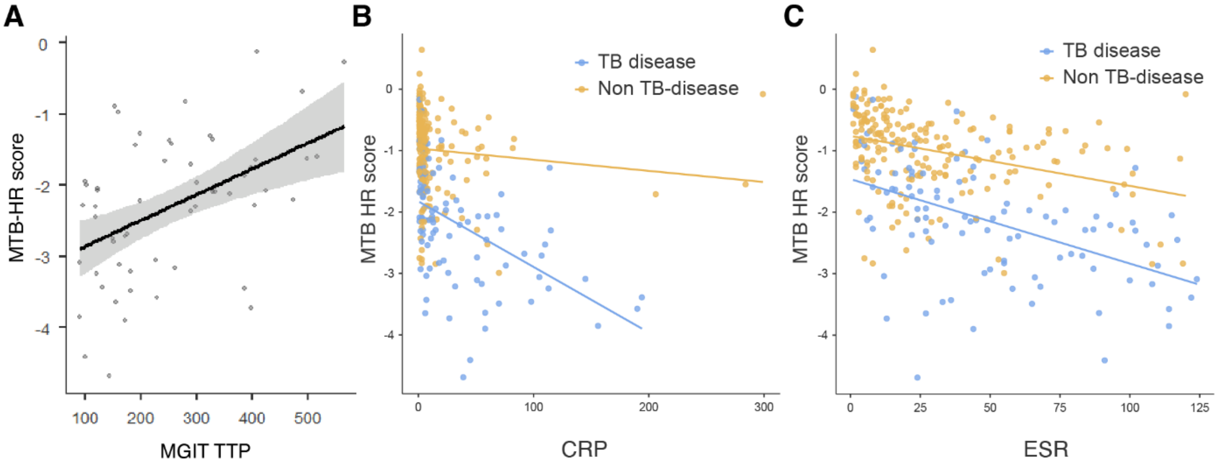

Figure S5 A) correlation MTB-HR score (Y-axis) and MGIT time to positive growth TTP (hours, X-axis) r = 0.463 (p<0.001), **B**) MTB-HR and CRP in TB disease, r= -0.499 (p<0.001) and in non TB disease r= -0.101 (ns), **C**) MTB-HR and ESR in TB disease r = -0.503 and in non TB disease r= -0.35 (p<0.001).

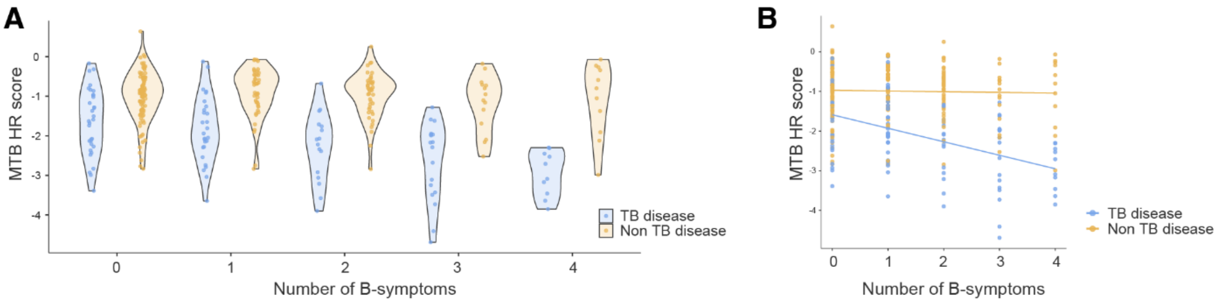

Figure S6. **A)** MTB HR score in relation to number of B-symptoms (fever, nightsweats, weight loss and diminished appetite) reported in TB disease and non TB disease. **B)** Correlation between MTB HR and number of B symptoms in TB disease r -0.284, p< 0.001, and non TB disease, no correlation.

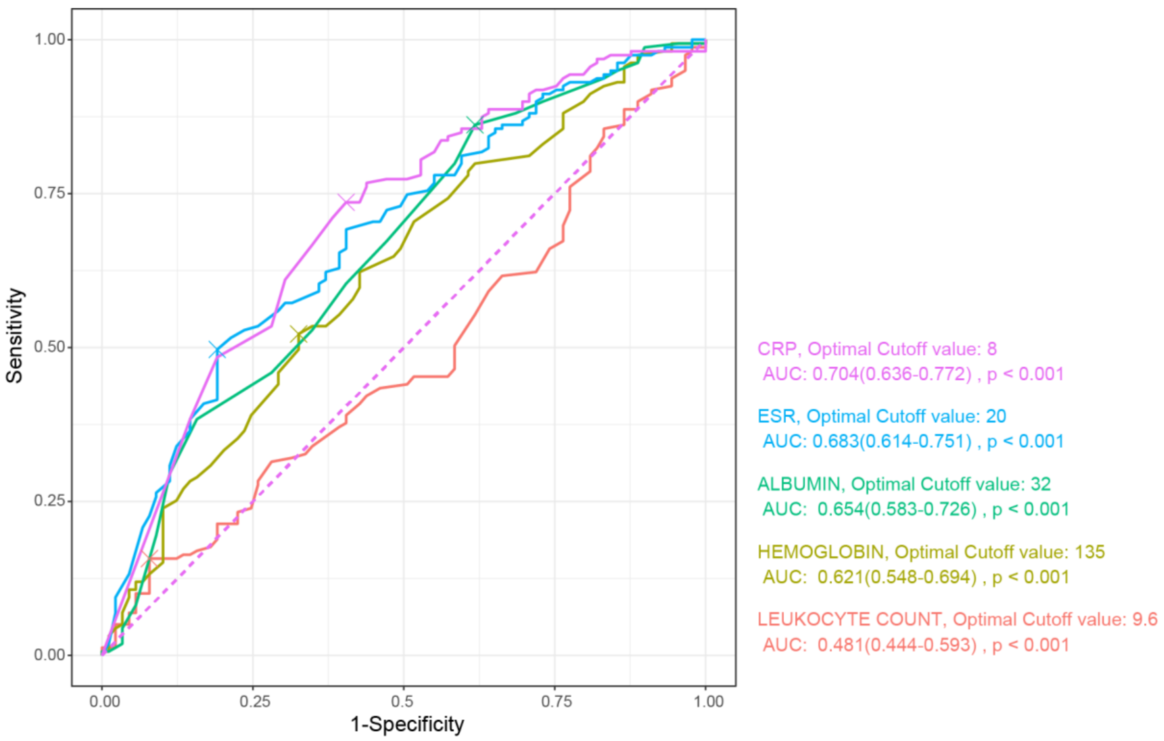

Figure S7. ROC-analysis of blood biomarkers CRP, ESR, Albumin, Hemoglobin and Leukocyte count for TB disease, AUC and Youden Index cut-off. CRP had the highest diagnostic accuracy for TB disease of the clinical biomarkers evaluated with an AUC of 0.70, cut-off value 8.

Supplemental Tables

Table S1. Clinical findings in Pulmonary TB, extrapulmonary TB and non-TB disease.

PATIENT GROUP		PTB n = 70	EPTB n = 38	All TB disease n = 108	Non TB disease n = 204
**ESR**	mm, ref < 20	52 (1-124)	37 (3-113) *	47 (1-124)	29 (1-120)**
**CRP**	mg/L, ref < 3	43 (1-194)	11 (1-76) *	32 (1-194)	14(1-299)**
**Hemoglobin**	g/L, ref >120(F)/>130(M)	128 (86-164)	132 (93-171)	129 (86-164)	135 (84-174)*
**Leukocyte count**	10^9/L, ref 4.4-10.0	7.4 (3.0-18.0)	6.1 (3.0-9.2) *	6.9 (3.0-18.0)	7.6 (0.7-155.1)
**Neutrophiles**	10^9/L, ref 1.6-5.9	4.4 (2.0-6.0)	3.3 (2.0-5.0)	4.5 (2.0-6.0)	4.3 (2.0-9.0)
**Albumin**	g/L, ref >38	32 (21-46)	37 (22-46)**	34 (21-46)	36 (18-45)*
**Fever**	Yes	32 (46)	5 (13)	37 (34)	65 (32)
**Loss of appetite**	Yes	27 (39)	5 (13)	32 (30)	36 (18)*
**Weight loss**	Yes	39 (56)	12 (32)	51 (47)	57 (28)**
**Night sweats**	Yes	31 (44)	9 (24)	40 (37)	59 (29)
**Fatigue**	Yes	33 (47)	10 (26)	43 (40)	45 (22)**
**Number of B-symptoms[1] n (%)**	0	14 (20)	21 (55)	35 (32)	91 (45)*
	1	17 (24)	10 (26)	27 (25)	45 (22)*
	2	14 (20)	2 (5)	16 (15)	43 (21)*
	3	16 (23)	3 (8)	19 (18)	14 (7)*
	4	9 (13)	2 (5)	11 (10)	11 (5)*
**Cough, n (%)**	Yes	56 (80)	6 (16)	62 (57)	135 (66)
**Chest Radiology n (%)**	Normal	0	18 (47)	18 (17)	70 (34)*
	Pathology CXR	44 (63)	5 (13)	49 (45)	70 (34)*
	Pathology CCT	26 (37)	13 (34)	39 (36)	60 (29)*
	Cavitation[2]	26 (37)	0	26 (24)	10 (5)
	Not done	0	2 (5)	2 (2)	4 (2)

[1]B-symptoms: fever, night sweats, weight loss and diminished appetite. Fatigue excluded as it was not consistently reported.
[2] Detected by CXR (9) and CCT (17) in pulmonary TB; CXR (5) and CCT (5) in non TB.
Biochemistry results presented as mean values (range). Independent samples Mann-U Whitney test was used to compare means. Chi^2 test used to compare proportions, Bonferri correction for multiple comparisons.
Pulmonary vs extrapulmonary TB, and TB disease vs non-TB disease * < 0.05; ** < 0.001.
CXR = chest Xray. CCT = Chest Computed Tomography

Table S2. MTB-HR score in TB and non TB and type of sample used in main analysis

MTB-HR score	TB disease n=104	PTB n=70	EPTB n=34	Non-TB disease n=204	Pulm (n=167)	Non Pulm (n=37)	TOTAL (n=308)
Mean (SD)	-2.09 (0.955)	-2.19 (1.03)	-1.90 (0.755)	-0.991 (0.650)	-0.975 (0.614)	-1.06 (0.797)	-1.36 (0.923)
Median (IQR)	-2.09 (1.33)	-2.13 (1.55)	-2.02 (1.08)	-0.870 (0.797)	-0.870 (0.772)	-0.937 (1.01)	-1.15 (1.29)
[Min, Max]	[-4.69, -0.124]	[-4.69, -0.124]	[-3.65, -0.360]	[-2.99, 0.640]	[-2.84, 0.250]	[-2.99, 0.640]	[-4.69, 0.640]
**Sample type pooled**							
Capillary	2 (1.9%)	1 (1.4%)	1 (2.9%)	3 (1.5%)	3 (1.8%)	0 (0%)	5 (1.6%)
Unassigned*	5 (4.8%)	5 (7.1%)	0 (0%)	11 (5.4%)	8 (4.8%)	3 (8.1%)	16 (5.2%)
venous	96 (92.3%)	63 (90.0%)	33 (97.1%)	190 (93.1%)	156 (93.4%)	34 (91.9%)	289 (93.8%)
Missing	1 (1.0%)	1 (1.4%)	0 (0%)	3 (1.5%)	0 (0%)	0 (0%)	1 (0%)

MTB-HR score in TB-disease; pulmonary (PTB) and extrapulmonary (EPTB) and non-TB disease; grouped by type of symptoms at inclusion (pulmonary and non-pulmonary), and type of patient sample used in the pooled analysis. *In 17 patients the samples could not with certainty be assigned as venous or capillary due to faulty registration in the computer software. One patient with TB disease had a non-valid venous analysis and no capillary sample leading to missing data. MTB-HR score; ATB vs non-TB: $p < 0.001$ PTB vs EPTB: $p=0.182$ Pulmonary vs non pulmonary non-TB $p= 0.594$. MTB-HR score = (CtGBP5+CtDUSP3)/2 – CtTBP

Table S3. TB and non-TB disease diagnosis groups and corresponding MTB HR TBP score

TB subgroup	N	MTB HR Mean	MTB HR Median	SD	MTB HR Minimum	MTB HR Maximum
**Pulmonary TB**						
Pulmonary TB	48	-2.138	-2.070	1.102	-4.691	-0.124
Pulmonary TB+pleuritis	6	-2.238	-2.135	0.502	-3.060	-1.710
Pulmonary+lymphnode TB	7	-1.790	-2.090	0.868	-2.930	-0.170
TB Pleuritis	2	-1.810	-1.810	0.750	-2.340	-1.280
Disseminated TB[1]	6	-3.178	-3.425	0.539	-3.575	-2.160
**Extrapulmonary TB**						
Lymphnode TB	27	-1.890	-2.076	0.749	-3.647	-0.360
Abdominal TB	3	-2.223	-2.830	0.627	-2.870	-0.970
Musculoskeletal TB	3	-2.030	-1.970	0.255	-2.310	-1.810
Ocular TB[2]	3	-0.767	-0.600	0.588	-1.420	-0.280
TB Otitis	1	-0.720	-0.720	N/A	-0.720	-0.720
**Non TB subgroup**	**N**	**MTB HR Mean**	**MTB HR Median**	**SD**	**MTB HR Minimum**	**MTB HR Maximum**
**Pulmonary conditions**						
Non-TB respiratory tract infection	52	-0.965	-0.820	0.613	-2.835	-0.0700
Bacterial	18	-1.045	-1.007	0.6555	-2.160	-0.0800
Viral	3	-1.041	-1.110	0.3325	-1.334	-0.6800
Nontuberculous mycobacterium	2	-1.050	-1.050	0.0849	-1.110	-0.9900
URTI	8	-1.016	-0.625	0.7197	-2.114	-0.3411
Unknown etiology	21	-0.857	-0.740	0.6156	-2.835	-0.0700
Non-TB respiratory tract infection + underlying lung disease	24	-1.109	-0.929	0.663	-2.833	-0.2208
Bacterial	10	-1.198	-1.161	0.6839	-2.527	-0.2700
Viral	2	-1.273	-1.273	0.2789	-1.470	-1.0755
Nontuberculous mycobacterium	2	-1.021	-1.021	0.9213	-1.673	-0.3700
Unknown etiology	10	-1.004	-0.825	0.7242	-2.833	-0.2208
Chronic lung diseases	34	-0.849	-0.845	0.441	-2.000	0.0400
Asthma	14	-1.003	-1.170	0.484	-1.620	0.2500
Inflammatory and Rheumatological conditions	13	-1.222	-0.970	0.554	-2.620	-0.6603
Upper airway conditions	12	-0.582	-0.590	0.338	-1.200	-0.0200
Sarcoidosis	10	-1.864	-1.936	0.844	-2.844	-0.2700
**Non pulmonary conditions**						
GI conditions	9	-0.940	-0.836	0.810	-2.250	-0.0700
Infection, non pulmonary[3]	8	-1.106	-0.825	0.936	-2.990	-0.1800
Benign adenopathy	7	-1.108	-0.980	0.706	-2.430	-0.4100
Malignancy	7	-0.850	-0.690	0.866	-2.160	0.6400
Urogenital condition	2	-0.449	-0.449	0.408	-0.737	-0.1600
No specific diagnose – healthy/symptoms resolved	12	-0.751	-0.730	0.565	-1.780	0.0000

Table S3. MTB-HR scores in TB disease and non-TB disease. MTB-HR score = (CtGBP5+CtDUSP3)/2 – CtTBP. [1]One patient with disseminated TB missing a valid MTB-HR result [2] Ocular TB clinical diagnosis, not included in ROC-analysis [3]Schistosomiasis (2); HIV; H pylori; Neoerlichios; Sialoadenitis with bacterial abscess; Scrotal abscess; Ileopsoas abscess (Serratia Marcescens).

Table S4. Xpert MTB-HR test performance in TB disease

Criterion	Cut-off	Sensitivity	Specificity	PPV	NPV
**All confirmed TB disease. AUC 0.832** (0.781-0.882)					
Youden Index	-1.575	0.718 (0.631-0.796)	0.843 (0.789-0.892)	0.698 (0.625-0.774)	0.856 (0.818-0.894)
Closest topleft	-1.505	0.728 (0.641-0.816)	0.833 (0.784-0.882)	0.69 (0.622-0.76)	0.859 (0.821-0.898)
TPP screening test minimum sensitivity	-0.86	**0.9**	0.5 (0.343-0.691)	0.476 (0.409-0.595)	0.908 (0.872-0.932)
TPP screening test minimum specificity	-1.23	0.825 (0.728-0.893)	**0.7**	0.581 (0.551-0.601)	0.888 (0.836-0.928)
TPP diagnostic test minimum overall sensitivity	-1.70	**0.65**	0.873 (0.784-0.932)	0.72 (0.603-0.827)	0.832 (0.816-0.841)
**Pulmonary TB overall. AUC 0.837** (0.776-0.898)					
Youden Index	-1.272	0.855 (0.768-0.928)	0.721 (0.657-0.779)	0.509 (0.45-0.571)	0.937 (0.899-0.969)
Closest topleft	-1.505	0.739 (0.638-0.841)	0.833 (0.779-0.882)	0.602 (0.523-0.682)	0.905 (0.87-0.94)
TPP screening test minimum sensitivity	-0.88	**0.9**	0.485 (0.093-0.747)	0.371 (0.251-0.546)	0.935 (0.734-0.957)
TPP screening test minimum specificity	-1.23	0.847 (0.739-0.928)	**0.7**	0.488 (0.455-0.511)	0.931 (0.888-0.966)
TPP diagnostic test minimum overall sensitivity	-1.75	**0.65**	0.882 (0.794-0.946)	0.651 (0.516-0.803)	0.882 (0.87-0.889)
Identified cut-off with sensitivity 90%[1]	-1.25	0.86	0.71	0.50	0.94
**Pulmonary TB Sputum PCR+ AUC 0.894** (0.841-0.948)					
Youden Index	-1.576	0.81 (0.69-0.929)	0.843 (0.794-0.887)	0.515 (0.433-0.603)	0.956 (0.929-0.982)
Closest topleft	-1.576	0.81 (0.69-0.929)	0.843 (0.794-0.892)	0.515 (0.438-0.613)	0.956 (0.929-0.983)
TPP screening test minimum sensitivity	-1.27	**0.9**	0.706 (0.444-0.863)	0.387 (0.25-0.574)	0.972 (0.956-0.977)
TPP screening test minimum specificity	-1.23	0.905 (0.786-0.976)	**0.7**	0.383 (0.35-0.401)	0.973 (0.941-0.993)

Criterion	Cut-off	Sensitivity	Specificity	PPV	NPV
**Pulmonary TB Sputum microscopy + AUC 0.939** (0.893-0.986)					
Youden Index	-1.98	0.88 (0.72-1)	0.912 (0.873-0.946)	0.55 (0.449-0.676)	0.984 (0.964-1)
Closest topleft	-1.98	0.88 (0.72-1)	0.912 (0.873-0.946)	0.55 (0.45-0.677)	0.984 (0.964-1)
TPP screening test minimum sensitivity	-1.43	**0.9**	0.813 (0.495-0.961)	0.371 (0.179-0.738)	0.985 (0.976-0.987)
TPP screening test minimum specificity	-1.23	0.96 (0.84-1)	**0.7**	0.282 (0.255-0.29)	0.993 (0.973-1)
**Extrapulmonary TB. AUC 0.821** (0.745-0.897)					
Youden Index	-1.575	0.706 (0.559-0.853)	0.843 (0.794-0.887)	0.431 (0.339-0.531)	0.945 (0.918-0.971)
Closest topleft	-1.575	0.706 (0.559-0.853)	0.843 (0.789-0.887)	0.431 (0.339-0.526)	0.946 (0.918-0.972)
TPP screening test minimum sensitivity	-0.86	**0.9**	0.51 (0.343-0.721)	0.234 (0.186-0.349)	0.968 (0.954-0.977)
TPP diagnostic test minimum sensitivity[2]	-1.65	**0.65**	0.853 (0.642-0.926)	0.424 (0.232-0.596)	0.936 (0.917-0.941)
TPP diagnostic test optimal specificity[3]	-2.76	0.147 (0.029-0.324)	**0.98**	0.551 (0.197-0.729)	0.873 (0.858-0.897)

Table S4. Test cut-offs with corresponding sensitivity, specificity, positive predictive value (PPV) and negative predictive value (NPV) for Youden Index, Topleft criterion and matched against WHO TPP criteria.[1]Suggested cut-off according to Gupta-Wright et al, (20) Lancet Glob Health 2024; 12: e226–34. [2]Minimal sensitivity requirements for culture confirmed TB overall, no minimum sensitivity target set for extrapulmonary TB. [3]98% specificity when compared against a microbiological reference standard.[4]
