# Supplementary material for: Improved Detection of Extrapulmonary and Paucibacillary Pulmonary Tuberculosis by Xpert MTB Host Response in a Tuberculosis Low-Endemic, High-Resource Setting
Source: J Infect Dis. 2025 Mar 6;232(1):e78–88. doi: 10.1093/infdis/jiaf110 (PMC12308647; doi:10.1093/infdis/jiaf110)
Supplement: jiaf110_Supplementary_Data [file jiaf110_supplementary_data.zip › Folkesson_Supplement_Inclusion_form.pdf]

Visit:
Visit 0

Demographics:

TB Status at Inclusion: ○ Suspected ○ Confirmed **[1]**

Date of Inclusion: ______ Now **[2]**

Year of Birth: ______ **[3]**

Sex: ○ Female ○ Male **[4]**

Country of birth: ______ **[5]**

Date of Immigration to Sweden if applicable: ______ **[6]**

Concurrent pregnancy? ○ Yes ○ No **[7]**

Length of Pregnancy (weeks): ______ **[8]**

Post partum period (6 months)? ○ Yes ○ No **[9]**

Delivery date: ______ **[10]**

Epidemiological:

Exposure to TB:

Known: ☐ **[11]** Date known: ______ **[12]**

Suspected: ☐ **[13]** Date suspected: ______ **[14]**

Previous TB:

Latent TB: ☐ [15]
Treated: ◯Yes ◯No [16]
Treatment date completed: [17]
PPD: ◯Positive [18] ◯Negative ◯Unknown
IGRA: ◯Positive [20] ◯Negative ◯Unknown
PPD date: [19]
IGRA date [21]

Active TB: ☐ [22]
Treated: ◯Yes ◯No [23]
Considered adequate treatment: ◯Yes ◯No [24]
Date treated: [25]
Country of treatment: [26]
Length of treatment (months): [27]

Active TB treatment completed: ◯Yes ◯No [28]
Date treatment completed: [29]

Comments:

[30]

Medical

Comorbidities:

HIV: ☐ [31]
Year of diagnosis: [32]
CD4 count: [33]
CD4 count date: [34]
Ongoing treatment: ◯Yes ◯No [35]
Viral load: [36]
Viral load date (most recent): [37]

Diabetes: ☐ [38]
Type: ◯Type 1 ◯Type 2 [39]
Latest HbA1c value (mmol/mol): [40]
Date HbA1c taken: [41]

Chronic kidney disease (GFR<60): ☐ [42]

Hemodialysis: ☐ [43]

Hematological disease: ☐ [44]
Ongoing/previous 5 years: [45]
Specify disease: [46]

Rheumatic disease: ☐ [47]
Ongoing/previous 5 years: [48]
Specify disease: [49]

Malignancy: ☐ [50]
Ongoing/previous 5 years: [51]
Specify disease [52]

Chronic lung disease: ☐ [53]
Specify disease: [54]

Comments:

[55]

Concomitant medication:

Oral cortisone: ☐ [56] | Drug being used: ____ [57] | Ongoing/last 6 months: ____ [58] | Current dose: ____ [59] | >15 mg prednisone for >1 month within last 6 months: ◯ Yes ◯ No [60]

TNF-alpha inhibtors: ☐ [61] | Drug being used: ____ [62] | Ongoing/last 6 months: ____ [63]

Other immunosuppressive med: ☐ [64] | Drug being used: ____ [65] | Ongoing/last 6 months: ____ [66]

Insulin: ☐ [67]

Cytotoxic chemotherapy: ☐ [68] | Drug being used: ____ [69] | Ongoing/previous 5 years: ____ [70]

Smoking:
◯ Yes ◯ No ◯ Ex-smoker [71]

Comments:

[72]

Clinical - Current Illness:

Symptoms that are the reason that the patient is investigated:

Start of symptoms: ____ Now [73]

General symptoms:
- ☐ Verified fever > 38 [74]
- ☐ Feeling feverish
- ☐ Night sweats
- ☐ Weight loss
- ☐ Loss of appetite
- ☐ Fatigue

Lung symptoms:
- ☐ Hemoptysis [75]
- ☐ Chest pain
- ☐ Shortness of breath
- ☐ Productive cough

cough ☐ [76]

Duration of cough:
- ◯ <1 week **[77]**
- ◯ 1-3 weeks
- ◯ 3 weeks - 2 months
- ◯ >2 months

Other symptoms:
- ☐ Localized back pain **[78]**
- ☐ Localized joint pain/swelling
- ☐ Lymphnode enlargement
- ☐ Abdominal pain/GI symptoms
- ☐ Headache
- ☐ Genitourinary symptoms

Neurological symptoms: ☐ **[79]**

If Neurological symptoms present, clarify:
- ☐ Seizures **[80]**
- ☐ Neurological deficit
- ☐ Impaired consciousness

Other neurological symptoms as applicable: ______ **[81]**

If other symptoms, specify: ______ **[82]**

Comments:

______ **[83]**

Weight (kg): ______ **[84]**

Laboratory results:

SR (mm) ______ **[85]**

CRP (mg/L) ______ **[86]**

Hemoglobin ______ **[87]**

Leukocyte count 10(9)/L ______ **[88]**

Neutrophil count 10(9)/L ______ **[89]**

Albumin (g/L ______ **[90]**

TB_Host_Response

Quantiferon gold: ○ Negative [91]
○ Positive
○ Indeterminate
○ Not available

Quantiferon date taken: [92]

Tuberculosis-Ag-1 (if QTF pos): [93]

Tuberculosis-Ag-2(if QTF pos): [94]

Radiology:

Chest Xray performed: ☐ [95]

Date: Now [96]

Lung X-Ray Normal ☐ [97]

Lung infiltration ○ Yes ○ No [98]

Unilateral or Bilateral: [99]

Cavitation ○ Yes ○ No [100]

Unilateral or bilateral [101]

Size [102]

One or multiple [103]

Pleaural Effusion ○ Yes ○ No [104]

Unilateral or bilateral [105]

Lymphadenopathy ○ Yes ○ No [106]

Fibronodular/granuloma ○ Yes ○ No [107]

Calcification ○ Yes ○ No [108]

Primary complex ○ Yes ○ No [109]

TB_Host_Response

Chest CT scan performed: [110] | Date ____ Now [111] | CT Normal [112]

Lung infiltration: Yes / No [113] | Unilateral or bilateral [114]

Cavitation: Yes / No [115] | Unilateral or bilateral [116] | Size ____ [117] | One or multiple [118]

Pleural effusion: Yes / No [119] | Unilateral or bilateral [120]

Lymphadenopathy: Yes / No [121]

Fibronodular/granuloma: Yes / No [122]

Tree-in-bud: Yes / No [123] | Unilateral or bilateral [124]

Calcification: Yes / No [125]

Primary complex: Yes / No [126]

Comments: ____ [127]

Abdominal CT:

Abdominal CT scan performed [128]

Date ____ Now [129]

Abdominal CT Normal [130]

Infiltration of liver or spleen [131]

Ascites [132]

Other [133]

Specify other if applicable: ____ [134]

TB_Host_Response

Brain CT/MRI:

Brain CT/MRI performed ☐ [135]

Date ____ Now [136]

Brain CT Normal ☐ [137]

Mass lesion ☐ [138]

Meningial enhancement ☐ [139]

Other ☐ [140]

Specify other if applicable: ____ [141]

Musculoskeletal CT/MRI

Musculoskeletal CT/MRI performed ☐ [142]

Date ____ Now [143]

Musculoskeletal Ct Normal ☐ [144]

Osteitis ☐ [145]

Spondylodiscitis ☐ [146]

Arthritis ☐ [147]

Abscesses ☐ [148]

Other ☐ [149]

Abscess locations if present: ____ [150]

Type/Location: ____ [151]

Specify other if applicable: ____ [152]

Comments: ____ [153]
